# Modelling the Impact of Cell-To-Cell Transmission in Hepatitis B Virus

**DOI:** 10.1371/journal.pone.0161978

**Published:** 2016-08-25

**Authors:** Ashish Goyal, John M. Murray

**Affiliations:** School of Mathematics and Statistics, UNSW, Sydney, NSW, Australia; Centre de Recherche en Cancerologie de Lyon, FRANCE

## Abstract

Cell-free virus is a well-recognized and efficient mechanism for the spread of hepatitis B virus (HBV) infection in the liver. Cell-to-cell transmission (CCT) can be a more efficient means of virus propagation. Despite experimental evidence implying CCT occurs in HBV, its relative impact is uncertain. We develop a 3-D agent-based model where each hepatocyte changes its viral state according to a dynamical process driven by cell-free virus infection, CCT and intracellular replication. We determine the relative importance of CCT in the development and resolution of acute HBV infection in the presence of cytolytic (CTL) and non-CTL mechanisms. T cell clearance number is defined as the minimum number of infected cells needed to be killed by each T cell at peak infection that results in infection clearance within 12 weeks with hepatocyte turnover (HT, number of equivalent livers) ≤3. We find that CCT has very little impact on the establishment of infection as the mean cccDNA copies/cell remains between 15 to 20 at the peak of the infection regardless of CCT strength. In contrast, CCT inhibit immune-mediated clearance of acute HBV infection as higher CCT strength requires higher T cell clearance number and increases the probability of T cell exhaustion. An effective non-CTL inhibition can counter these negative effects of higher strengths of CCT by supporting rapid, efficient viral clearance and with little liver destruction. This is evident as the T cell clearance number drops by approximately 50% when non-CTL inhibition is increased from 10% to 80%. Higher CCT strength also increases the probability of the incidence of fulminant hepatitis with this phenomenon being unlikely to arise for no CCT. In conclusion, we report the possibility of CCT impacting HBV clearance and its contribution to fulminant hepatitis.

## Introduction

Hepatitis B virus (HBV) is a leading cause of liver cancer around the world. Although several treatment options such as lamivudine, entecavir and peg-Interferon exist, their efficacy is limited to around 65–70% [1]. With 400 million chronic infections worldwide and millions newly infected each year, it is important to improve the efficacy of these therapies. Approximately 5–10% of children and 90–95% of adults resolve acute HBV infection through their immune response. Understanding and differentiating the factors which determine a successful immune clearance may lead to enhancement in the efficacy of antiviral therapies. How immunological mechanisms fail and result in viral persistence is also not well understood [2].

The spread of HBV infection in the liver is mainly due to cell-free virus [3]. However HBV viral infection is also found to be more favourable to cells adjacent to infected cells as spatial clusters of infected hepatocyte were found during early phases of infection [3]. The existence of these clusters was attributed to the polarized egress of virions in infected hepatocytes [3]. However recent advancements show that along with polarization, high surface retention and cell adhesion of hepatocytes cause double envelope virions such as HBV to support cell-cell fusion, a form of cell-to-cell transmission (CCT) [4, 5]. The emergence of clusters could also be due to infection by virions exported from a neighbouring infected cell, although this cannot be discriminated from CCT in regards to mathematical modelling. The capability for exosomes and synapses to transmit infection also indicates a strong possibility of CCT in intrahepatic HBV spread [4–9]. Other viruses such as human immunodeficiency virus (HIV) and hepatitis C virus (HCV) have already been found to use CCT [5, 8, 10–12]. The spread of infection through free virus is an important route of transmission for any virus but CCT has its own advantages such as fewer biophysical and kinetic limitations, and possible evasion from the immune system [8, 12]. In the past, dynamic HBV models have only considered cell-free virus as responsible for viral spread [1, 13–19]. The relative contribution of CCT in HBV infection and persistence are poorly understood, and will be investigated through modelling in this manuscript.

The role of the innate immune response in the resolution of HBV acute infection has been controversial [20, 21]. Recent studies have suggested that it is more likely to be on the weaker side [21, 22] whereas the adaptive immune response is considered mainly responsible for the clearance of HBV acute infection [21]. It is evident that both cytolytic (CTL) and non-CTL mechanisms are an essential part of adaptive immune-mediated clearance. CTL action kills infected cells while non-CTL effects induce intracellular inhibition of viral replication [13, 15, 23, 24]. Additional factors such as the loss of intracellular HBV intermediates during cell proliferation also help clear infection incurring between 0.7 and 3 complete hepatocyte turnovers (HT) within 12 weeks [13]. These studies have significantly improved our understanding of immune-mediated clearance of acute HBV infection. However, CCT if supported by HBV, can also potentially impact the relative contribution of these mechanisms which are responsible for acute infection clearance. We also investigate the impact of CCT on clearance of infection.

Mathematical models have proven useful in determining the characteristics of several viruses including HBV [1, 13–15, 17–20, 25]. However these models generally assume a single compartment for the liver where infection spreads homogeneously. Since our investigations concern the impact of an infected cell’s transmission to its neighbours possibly through CCT, the model developed here incorporates the (simplified) 3D structure of the liver. As in Murray & Goyal [13], each cell is placed at a lattice point of a 3D cube and behaves as an individual agent. A cell changes its state according to a dynamical process driven by cell-free virus infection, CCT and intracellular replication. This spatial intrahepatic 3D model of HBV infection allows the study of the processes that can contribute to the development of clusters and the heterogeneous spread of infection in the liver. It additionally allows the investigation of the case when the CTL component of the adaptive immune response acts at a local level (moving from one infected cell to its neighbour). It further allows us to investigate whether the CTL component chooses infected cells with higher cccDNA content or randomly. Our results indicate CCT does not greatly impact the establishment of infection but hinders immune-mediated clearance.

## Materials and Methods

**Fig 1:**
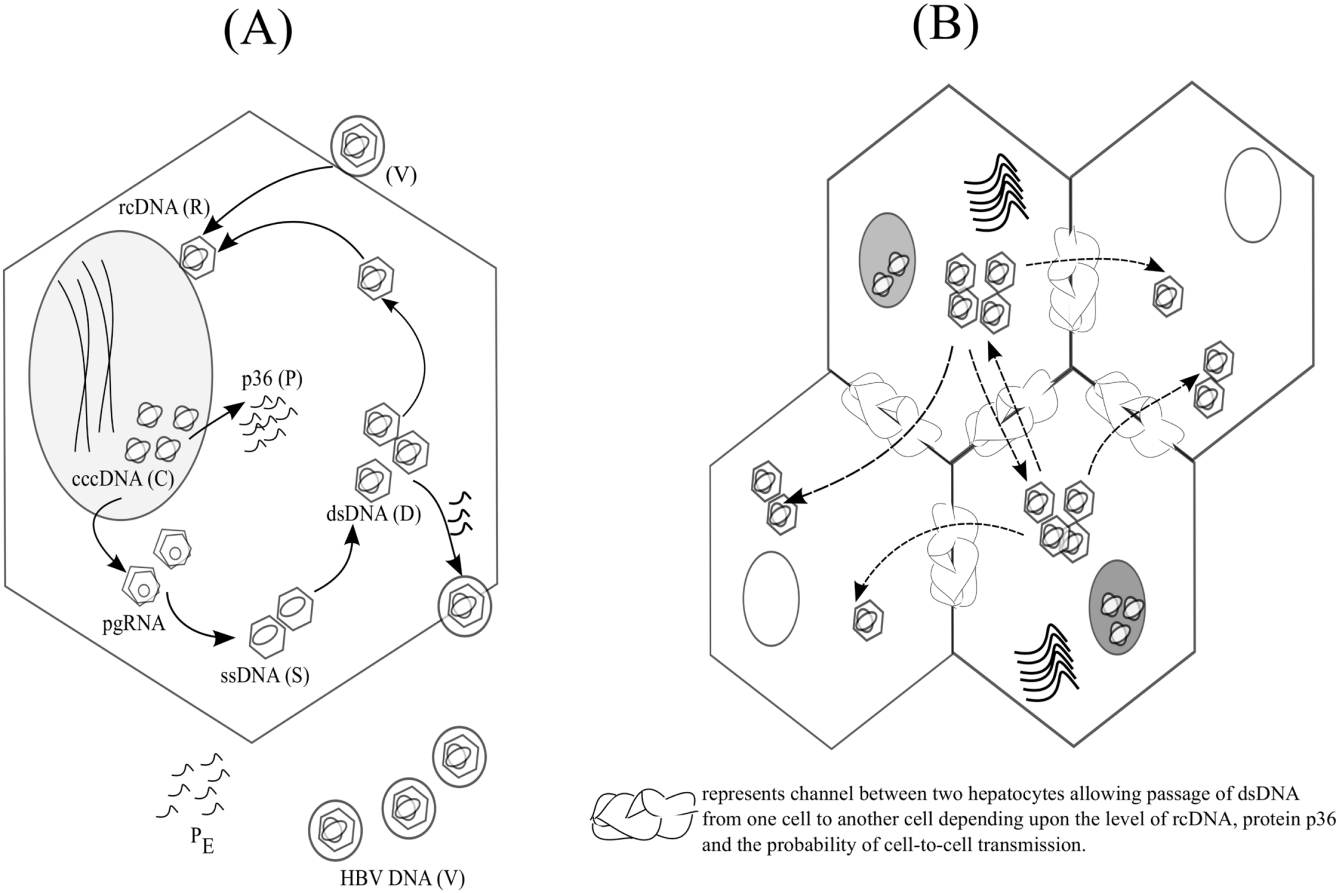
HBV replication and cell-to-cell transmission. (A) The HBV life cycle: a HBV DNA virion (*V*) enters a hepatocyte to deliver rcDNA (*R*). This is transferred to the nucleus where it is repaired to form cccDNA (*C*), the template of HBV infection. Transcription of cccDNA produces pgRNA which after reverse transcription forms ssDNA (*S*) and dsDNA (*D*). Depending on the level of large surface antigen (*P*), these dsDNA are either transported back to the nucleus or are exported as new HBV virions. (B) Cell-to-cell transmission between hepatocytes: depending on the level of dsDNA and large surface antigen, infected cells can spread HBV to neighbouring cells. We consider the separate CCT possibilities that an infected cell only transmits infection to uninfected cells or whether it also can increase the rcDNA content in previously infected cells. Transmission to previously infected cells can lead to an increase in cccDNA content.

HBV infection is initiated by virion (*V*) attachment to an hepatocyte. After entering the hepatocyte, the virion uncoats to relaxed circular DNA (rcDNA, *R*). These rcDNA are then transferred to the nucleus which after repair are established as the template of HBV infection, covalently closed circular HBV DNA (cccDNA, *C*). These cccDNA transcribe pregenomic RNA (pgRNA) which upon reverse transcription produce single stranded DNA (ssDNA, *S*), and double stranded DNA (dsDNA, *D*). Although dsDNA are equivalent to relaxed circular DNA (rcDNA), we characterize them separately to distinguish newly infecting virions from products of intracellular replication [13]. CccDNA in the nucleus also produce small, medium and large envelope proteins (*P*). The levels of *P* determine whether to export dsDNA as virions or to redirect them to the nucleus to increase cccDNA copy number. These proteins are also exported as *P*_*E*_. The basics of infection in a single hepatocyte are shown in Fig 1(A).

The differential equation model of intracellular replication is derived from Murray and Goyal [13].

$$\begin{array}{ll} \dot{R} & =kV-\mu_{R}R-be^{\left(-\lambda P\right)}R\\ \dot{C} & =be^{\left(-\lambda P\right)}[D+R]-\mu C\\ \dot{S} & =aC-bS\\ \dot{D} & =bS-bD\\ \dot{P} & =a_{P}C-bP\\ {\dot{P}}_{E} & =bP-cP_{E}\\ \dot{V} & =b\left(1-e^{\left(-\lambda P\right)}\right)D-cV \end{array}$$ (1)

All parameters in model (1) are described in Table 1.

**Table 1:** Parameters, their description and values in model (1) of a single infected cell.

Parameter	Description	References
*k*	Virion infectivity	0.03 [13]
*μ*_*R*_	Removal rate of infecting rcDNA	$\frac{\text{log }2}{1}$ day^-1^ [13]
*b*	Feedback rate of infecting rcDNA, and export rate of protein p36 and virions	$\frac{\text{log }2}{1}$ day^-1^ [13, 18]
*λ*^−1^	Average p36 level	100,000 molecules per cell [13, 26]
*μ*	Half-life of cccDNA	$\frac{\text{log }2}{50}$ day^-1^[13, 27]
*a*	Production rate of ssDNA from cccDNA	50*b* day^-1^ [13, 28]
*a*_*P*_	Production rate of p36 protein from cccDNA	1000*a* day^-1^ [13]
*c*	Decay rate of exported proteins and virions	$\frac{\text{24}\times \text{log }2}{4}$ day^-1^ [13, 18]
*N*	Number of hepatocytes in the simulation	2.7×10^4^ (2.7×10^4^ − 10^6^)
*γ*	Cell-to-cell transmission probability	(0 − 1) (Assumed)

### Stochastic agent based model

The differential equation model (1) describes infection within a single cell. However, we also want to evaluate infection between cells. Therefore, we consider an agent based model of *N* cells placed in a 3-D cubic lattice. A cell can be infected by one of its six neighbour cells (Fig 1B) through CCT as well as from circulating virions.

### Intracellular replication

First we discretize the time interval for the stochastic process into a step size of *tstep* (= 0.01 days) [13]. The differential equation dynamics of all *N* hepatocytes (*i* = 1,…, N) are converted into discrete stochastic dynamics by using a Poisson distribution about the mean values given in Table 1. The mathematical equivalent of the stochastic model for hepatocyte ‘*i* ‘ at time ‘*t+1*’ is described by [13],

$$\begin{array}{ll} R_{t+1}^{i} & =R_{t}^{i}+Poisson[(kV_{t}-\mu_{R}R_{t}^{i}-be^{\left(-\lambda P_{t}^{i}\right)}R_{t}^{i})tstep]\\ C_{t+1}^{i} & =C_{t}^{i}+Poisson[(be^{\left(-\lambda P_{t}^{i}\right)}[D_{t}^{i}+R_{t}^{i}]-\mu C_{t}^{i})tstep]\\ S_{t+1}^{i} & =S_{t}^{i}+Poisson[((1-\text{u})aC_{t}^{i}-bS_{t}^{i})tstep]\\ D_{t+1}^{i} & =D_{t}^{i}+Poisson[(bS_{t}^{i}-bD_{t}^{i})tstep]\\ P_{t+1}^{i} & =P_{t}^{i}+Poisson[(a_{P}C_{t}^{i}-bP_{t}^{i})tstep]\\ P_{E_{t+1}}^{i} & =P_{E_{t}}^{i}+Poisson[(bP_{t}^{i}-cP_{E_{t}}^{i})tstep]\\ V_{t+1} & =V_{t}+\sum_{i}Poisson[(b(1-e^{\left(-\lambda P_{t}^{i}\right)})D_{t}^{i}-cV_{t})tstep] \end{array}$$ (2)

Here *Poisson*(*χ*) generates a random number satisfying a Poisson distribution with mean value *χ*. In addition, we model a non-CTL immune response which suppresses the production rate of ssDNA by a factor *u*(0≤*u*≤1) in all infected hepatocytes. Note that *V* represents the total pool of virions rather than the output from any single cell.

### Cell-to-cell transmission

As well as the stochastic model of infection from circulating virions, intracellular replication and viral production given by the set of Eq (2), we additionally considered CCT between neighbouring cells. Since CCT is dependent on the local concentration of dsDNA and protein levels [3, 29], we model it through the addition of the term *γb*(1−e^−*λP*^)*D* in the equation of $R_{t+1}^{i}$, where *γ* represents the strength of CCT from a cell to its neighbouring cells. We also represent *B*^*i*^ as the set of all 6 neighbour cells of hepatocyte ‘*i*’. Therefore after incorporating CCT, the first equation in model (2) for hepatocyte ‘*i*’ becomes

$$R_{t+1}^{i}=R_{t}^{i}+Poisson[(kV_{t}-\mu_{R}R_{t}^{i}-be^{-\left(\lambda P_{t}^{i}\right)}R_{t}^{i})tstep]+\sum_{j\in B^{i}}Poisson[(\gamma b(1-e^{-\lambda P_{t}^{j}})D_{t}^{j})tstep]$$

In order to preserve quantities, we subtract the amount of dsDNA exported as rcDNA to hepatocyte ‘*i*’ from neighbours *j*∈*B*^*i*^ through CCT as follows,

$$D_{t+1}^{j}=D_{t}^{j}+Poisson[(bS_{t}^{j}-bD_{t}^{j})\text{tstep}]-Poisson[(\gamma b(1-e^{-\lambda P_{t}^{j}})D_{t}^{j})tstep]$$

Similarly, the final equation in model (2) is modified as,

$$V_{t+1}=V_{t}+\sum_{i}Poisson[(b\left(1-\gamma \right)(1-e^{\left(-\lambda {\text{P}}_{t}^{i}\right)})D_{t}^{i}-cV_{t})tstep]$$

Since there were limited data that quantified the contribution that CCT makes to HBV infection, we tested four levels of CCT:

1. *γ* = 0,
2. *γ* = 0.0002,
3. *γ* = 0.009, and
4. *γ* = 0.2.

In this manuscript, we will refer to *γ* = 0, *γ* = 0.0002, *γ* = 0.009 and *γ* = 0.2 as no CCT, weak CCT, moderate CCT and strong CCT respectively. The values of *γ* were chosen to produce different rates of the spread of infection within the liver satisfying two criteria, (i) spatial clusters are observed between week 1 and 2 as found experimentally [3], and (ii) the contribution of amplification in the cccDNA accumulation is 90% or higher as found in duck experiments [26].

### Hepatocyte natural death

At each timestep, we choose hepatocytes $i\in I_{D}=B(\text{N},\frac{180}{tstep})$ for natural death using a binomial distribution such that the average life of a hepatocyte is 6 months [13].

### Hepatocyte cytolytic killing through the adaptive immune system

It is evident that CTL and non-CTL mechanisms are an essential part of the adaptive immune response so both of these processes are included in all simulations [13, 23]. Approximately 2×10^8^ CD8+ T cells are activated for a full liver size every day [30–32]. This number of activated T cells is very small compared to the approximately 10^11^ hepatocytes in a liver which can be completely infected at the peak of infection [21]. Therefore we define the T cell response (*n*_*R*_) as the number of infected cells killed by each T cell on average per day at the peak of infection (and this response has been found to remain constant thereafter [33]). We additionally define the T cell clearance number (*n*_*CTL*_) as the minimum T cell response that can ensure the immune system clears acute infection (assumed to be complete by day 45) clearance within 12 weeks and with HT<3, as found experimentally [13, 23, 24, 34]. We also define infection clearance as achieving zero cccDNA and no virions in the simulation.

**Fig 2:**
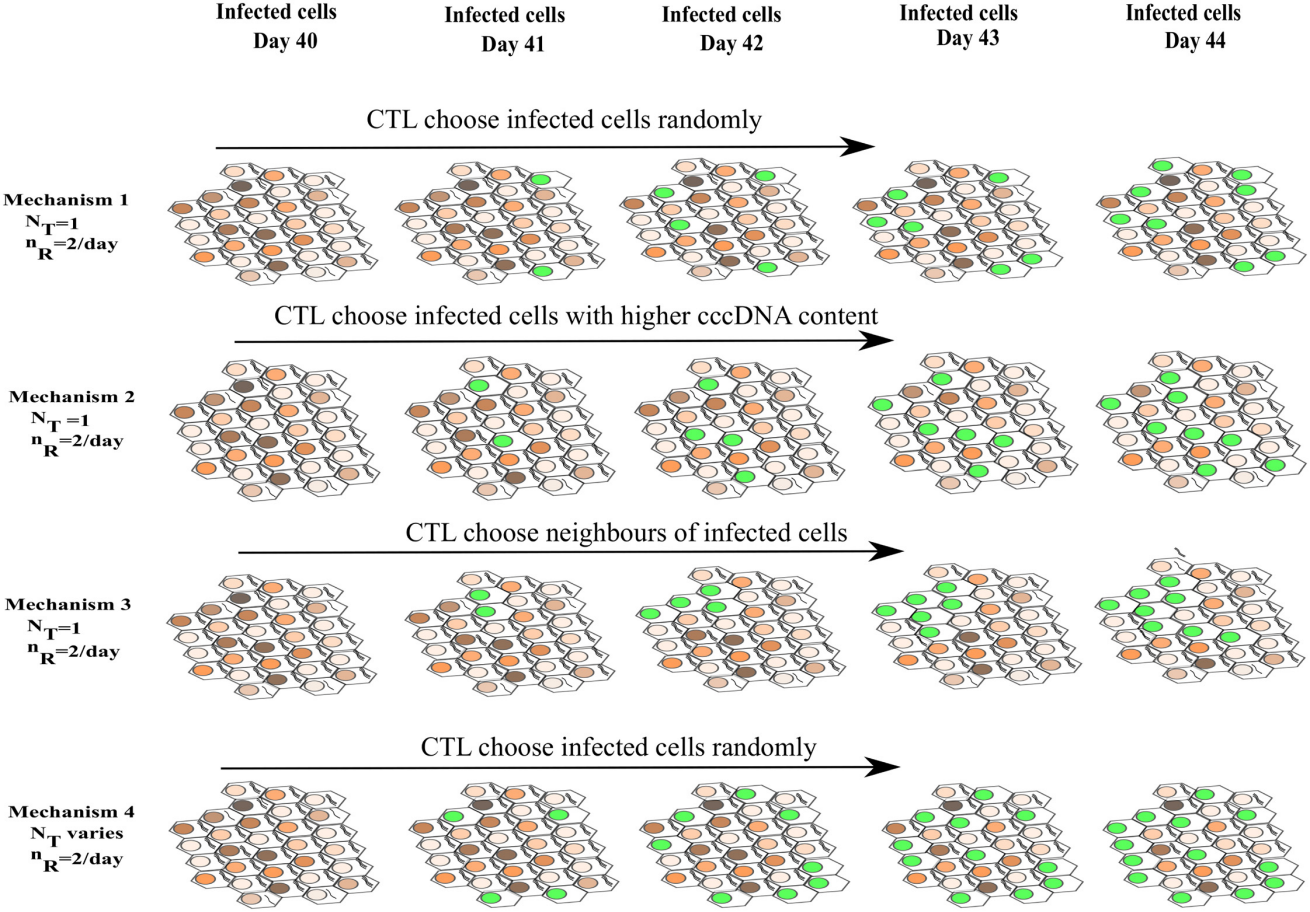
Four possible scenarios describing CTL removal of infected cells. In the figure, the cell (represented by a hexagon) with the highest cccDNA content in the nucleus (represented by an ellipse) is shown with the darkest shade of brown. A nucleus coloured green represents the pattern of infected cells killed by CTL under each mechanism. The first three mechanisms remove a constant number of infected cells (*n*_*R*_×*N*_*T*_) per day regardless of the number of infected cells, whereas the clearance rate of infected cells by mechanism 4 increases with an increase in the number of infected cells. All four mechanisms assume that there are *N*_*T*_ HBV-specific CTLs after day 45 when the adaptive immune system is assumed to commence. Mechanism 1 kills infected cells randomly; mechanism 2 prioritises infected cells with the highest cccDNA content while mechanism 3 first kills a cell with the highest cccDNA content followed by one of its infected neighbours, also with the highest cccDNA content and so on. So mechanism 3 results in CTL following a path through an infected cluster. On the other hand, mechanism 4 assumes that the T cell clearance number varies relative to the infection level, killing infected cells randomly.

It has been found that the number of HBV-specific CD8+ T cells varies with the number of HBV infected cells in the liver [13, 33, 35]. However, experimental studies in chimpanzees revealed that the total CD8+ T cell count can be stable (which usually leads to constant intrahepatic HBV DNA content) [36]. Using the concept of T cell clearance number, we explore the dynamics of HBV infection under two scenarios where activated HBV-specific CD8+ T cell numbers for a full liver size either (i) stay at a constant level (2×10^8^ T cells every day [30–32]), or (ii) change according to the number of infected cells. Since our simulations only incorporate *N* hepatocytes compared to a real liver with approximately 6×10^10^ hepatocytes, we scale the number of available HBV-specific CD8+ T cells as $N_{T}=[(\frac{2\times 10^{8}\times N}{6\times 10^{10}})]$ in our simulations. We investigate four scenarios under which CTL can target and remove infected cells (Fig 2),

1. Mechanism 1 (M1): In this case, at each time step *Poisson*(*N*_*T*_×*n*_*R*_×*tstep*) infected cells are selected randomly for cytolytic removal.
2. Mechanism 2 (M2): At each time step all infected cells are first sorted by their cccDNA levels (in the order of highest to lowest) and then the top *Poisson*(*N*_*T*_×*n*_*R*_×*tstep*) infected cells are selected for cytolytic killing.
3. Mechanism 3 (M3): At each time step all infected cells are first sorted by their cccDNA levels (in the order of highest to lowest) and the first *N*_*T*_ of these are chosen to be the first cleared by CTL in that time step. Thereafter, for each of these *N*_*T*_ cells, an infected neighbouring cell with the highest cccDNA number is selected for cytolytic killing and so on. If a CTL reaches a point where there are no infected neighbours, then it moves to another cluster and repeats the same process. This process is repeated until *Poisson*(*N*_*T*_×*n*_*R*_×*tstep*) infected cells are killed.
4. Mechanism 4 (M4): In this case, the T cell response is adaptive, varying according to the level of infection as previously proposed by Murray et al. [13]. We model a CTL response killing a set of infected cells $i\in I_{CTL\;}=B(\text{n},1-{\text{e}}^{\left(-\delta \frac{tstep}{1+\frac{n}{N}}\right)})$ at each time step through a binomial distribution dependent on the number of infected hepatocytes *n* and the total number of hepatocytes in the simulation *N*. The parameter *δ* (day^-1^) reflects the strength of the CTL response. The survival probability of an infected cell increases with the number of infected hepatocytes *n* due to a limited capacity of CTL clearance [24].

To test sensitivity of our results with respect to the number *N*_*T*_ of CTL, simulations of these clearance mechanisms are repeated under the assumption that every day a 10-fold higher number of 2×10^9^ T cells are activated for a full liver size.

### Hepatocyte proliferation to maintain constant liver mass

We maintain a constant number of *N* hepatocytes in these simulations, through compensatory cell proliferation. Hepatocytes, both infected and uninfected, are randomly chosen through a binomial distribution to undergo proliferation in relation to the proportion of hepatocytes lost at each time step. When a cell dies, one of its neighbour cells is randomly chosen for cell proliferation. The neighbour cell could be infected or uninfected, but in both cases two uninfected daughter cells are assumed to be produced [13].

### Simulations

Model (2) is first simulated in MATLAB R2012a for 45 days (between 6 to 8 weeks) to allow the establishment of peak infection in the acute phase while assuming no adaptive immune response (*u* = 0, and no immune-mediated CTL killing of hepatocytes) [34]. After day 45, the immune system is assumed activated (*u*≠0 and *CTL*≠0) and the model is simulated for a further 12 weeks to track clearance.

We convert total body load of HBV DNA and HBsAg to copies/mL by assuming 3L of serum in the average human through which virions are distributed. We also scale serum levels for this much smaller number of hepatocytes *N* = 2.7×10^4^ in our simulations, to what would be expected from a liver containing approximately 6×10^10^ hepatocytes, by scaling the relative proportions of each. Hence the number of virions in serum calculated from these simulations *V* is converted to a human equivalent per mL of serum $\hat{V}$ through the formula

$$\hat{V}=V\times \frac{6\times 10^{10}}{N}/3000$$ (3)

### Procedure

The CTL strength of the adaptive immune response through mechanisms M1, M2 and M3 is varied by changing *n*_*R*_ while M4 is modified through *δ*. Non-zero positive values of *n*_*R*_, *δ* and *u* [36] are investigated in multiples of 5, 0.05 and 0.1 respectively.

We first report *n*_*CTL*_ under M1 for a particular degree of non-CTL inhibition (*u*) such that infection is cleared. We then employ the same *n*_*CTL*_ value for M2 and M3 to compare their performances with M1. Each experiment with the stochastic model is repeated 100 times and then the mean [min max] for both HT and time at which viremia achieves less than 1 copy in the body (viremia clearance time, *t*_*v*_) is reported.

## Results

We first investigate the establishment of infection and assume the adaptive immune response against HBV has not commenced. The inclusion of the 3D structure of infection allows us to determine the feasibility of CCT as an important route of the spread of HBV infection.

### HBV is likely to exhibit cell-to-cell transmission

We initially investigate whether CCT assists the establishment of HBV acute infection under two scenarios, (i) CCT is only allowed from an infected to an uninfected cell, and (ii) CCT is allowed between two infected cells as well so that it may increase infection levels within a previously infected cell.

In *in vitro* experiments, Funk et al. found spatial clusters (close groups of infected hepatocytes at different locations in the liver) during early phases of HBV replication [3]. In our simulations under both scenarios, spatial clusters are observed between week 1 and 2 only when CCT also acts as a mechanism of HBV spread along with cell-free virus infection (shown for day 12 with moderate CCT in S1 and S2 Figs). Thus CCT is likely to be an essential part of HBV infection spread in the liver. In the presence of moderate CCT, we observe 63 clusters of infected cells of size 2 to 29 relative to the total number of 30^3^ = 27,000 hepatocytes at day 12. As the CCT strength increases, the size of the largest cluster increases. For example, in the presence of high CCT we observe 148 clusters with size varying between 2 and 582 infected cells.

Summers et al. determined that over 90% of cccDNA accumulation in ducks is a result of amplification (transportation of rcDNA back to the nucleus) [26]. Our results (irrespective of CCT strength) are consistent with this observation only when CCT is not allowed between two infected cells (S1 Table). If CCT is additionally allowed between two infected cells, then the contribution of amplification only results in approximately 76% of cccDNA arising through accumulation. These results are in line with the Mothes et al. observation that enveloped viruses mainly use CCT as a mechanism to transmit infection from infected to uninfected cells and once the virus is transmitted, cell-cell contacts are downregulated [8]. Therefore CCT between cells that are already infected is unlikely and the remaining experiments are performed assuming CCT can only occur between an infected and an uninfected cell.

**Fig 3:**
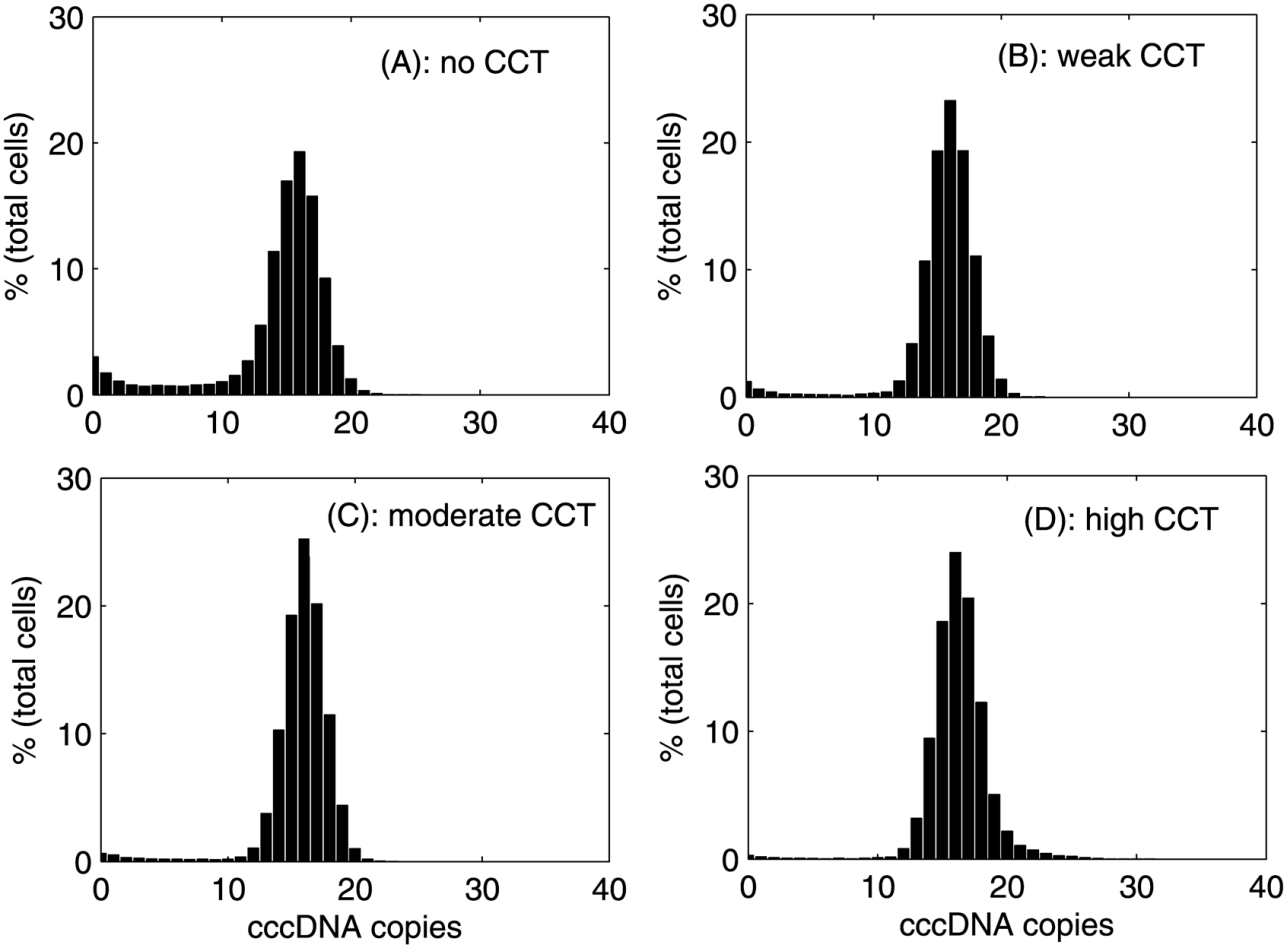
Change in the distribution of cccDNA copies/cell with CCT strength. Distribution of cccDNA copies per cell at day 45 under varying strengths of CCT assuming no cell-to-cell transmission between cells that are already infected, (A) no CCT, (B) weak CCT, (C) moderate CCT, (D) high CCT.

Regardless of variation in CCT strength (*γ*), the mean number of cccDNA remains between 15 and 20 copies/cell at peak infection (S3 Fig and [13]). Additionally cccDNA copies per cell at day 45 are normally distributed with almost 90% of the nuclei containing between 1 and 17 cccDNA copies irrespective of the choice of CCT strength (Fig 3), which is in line with findings for duck HBV [37].

### Lattice size does not substantially impact on simulations of the establishment of acute infection

Computation with the same number of cells as in a real liver is infeasible so we check whether the lower number of cells included in our simulations can greatly impact results. Different lattice sizes with 30^3^ = 27,000 and 100^3^ = 10^6^ hepatocytes have no impact on either the mean or the distribution of cccDNA copies/cell under all CCT strengths (Fig not shown). In all cases, almost 95% to 99% of hepatocytes are infected at peak infection. The time to reach peak infection increases slightly by 2 to 5 days when we increase lattice size; however this is still in the range of 6 to 8 weeks. Hence acute infection simulations with 27,000 hepatocytes should reasonably represent the case for a full sized liver.

### Lattice size only impacts simulations of the clearance of acute infection under cytolytic killing mechanism M3

We assume the adaptive immune response begins at the peak of infection when almost all cells in the liver are infected. The CTL killing mechanisms M3 (movement of a T cell from an infected cell to another infected cell in the neighbourhood) and M4 (adaptive immune response varying with the level of infection) are more likely to represent the actual biological processes compared to M1 and M2 that assume a CTL can instantly move between different regions of the liver [35, 38]. Although M1 and M2 are unreasonable in that sense, they are included here as they provide bounds on clearance of the more complex process in M3, such that the amount of cccDNA cleared in each step will be given by M2≤M3≤M1. Nevertheless, we are interested in determining whether simulations with 27,000 hepatocytes can reasonably reflect the process of acute infection clearance for a full size liver under M3 and M4.

**Fig 4:**
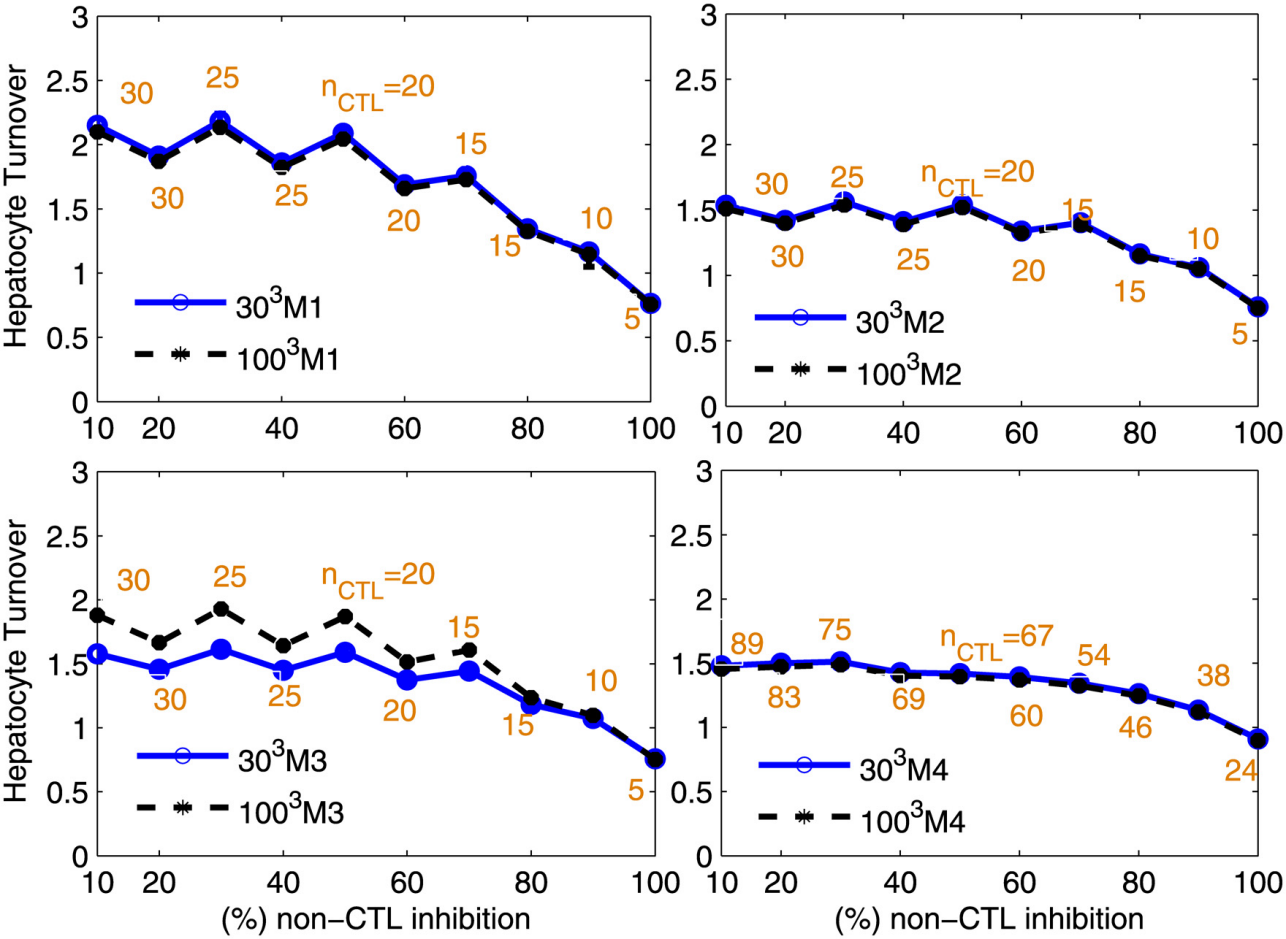
Relation between different CTL mechanisms, non-CTL inhibition and HT during infection clearance. Simulations were performed with weak CCT and under different lattice sizes to determine its impact. The T cell clearance number (*n*_*CTL*_) is shown as brown text at each data point under the different mechanisms of cytolytic killing, (A) Mechanism 1 (M1), (B) Mechanism 2 (M2), (C) Mechanism 3 (M3), and (D) Mechanism 4 (M4). In the figure legends, 30^3^M1 and 100^3^M1 refer to simulations conducted with *N* = 30^3^ and *N* = 100^3^ hepatocytes under mechanism 1. The higher is non-CTL inhibition the fewer CTLs are required to clear infection as represented by lower *n*_*CTL*_. As non-CTL inhibition increases in strength, the number of cells required to be killed by each CTL (*n*_*CTL*_) to achieve clearance either remains constant or decreases. For a given non-CTL inhibition, an *n*_*R*_ higher than *n*_*CTL*_ will also clear infection with lower HT. The peaks are an artefact of restricting calculations of *n*_*CTL*_ to multiples of 5 for M1, M2 and M3.

Under clearance mechanism M4, neither the amount of hepatocyte turnover (HT) nor the T cell clearance number is substantially affected by lattice size regardless of CCT (without CCT: S2 and S3 Tables, and with CCT: Fig 4).

On the other hand, we observe an increase in HT but no impact on T cell clearance number under M3 as a result of an increase in lattice size (Fig 4, S4 and S5 Tables). For example, mean HT (*t*_*v*_) are 1.8 (25) and 2.5 (25) for simulations with 30^3^ = 27,000 and 100^3^ = 10^6^ hepatocytes respectively at 10% non-CTL inhibition. This is because restriction of the movement of a T cell from an infected cell to one of its neighbour cells (that might be only recently infected with few cccDNA copies), can allow more highly infected cells outside of that cluster to remain. Therefore, it is understandable that simulations of the clearance of acute infection under M3 with 27,000 cells may not be valid for a full liver size. However since M1 and M2 provide bounds on clearance and related factors (M2≤M3≤M1), we instead use their values as estimates for the impact of M3 with larger lattice sizes (Fig 4 and S4 Fig). For example, mean HT is 2.6, 1.8 and 1.8 at 10% non-CTL inhibition under M1, M2 and M3 respectively for simulations with 30^3^ = 27,000 hepatocytes while it is 2.6, 1.8 and 2.5 for simulations with 100^3^ = 10^6^ hepatocytes. In particular, we find that HT under M3 is comparable to HT under M1 for a large lattice size. Therefore, infection clearance under M1 reasonably reflects infection clearance under M3. Hereafter, we will use M1 to provide estimates for M3.

**Fig 5:**
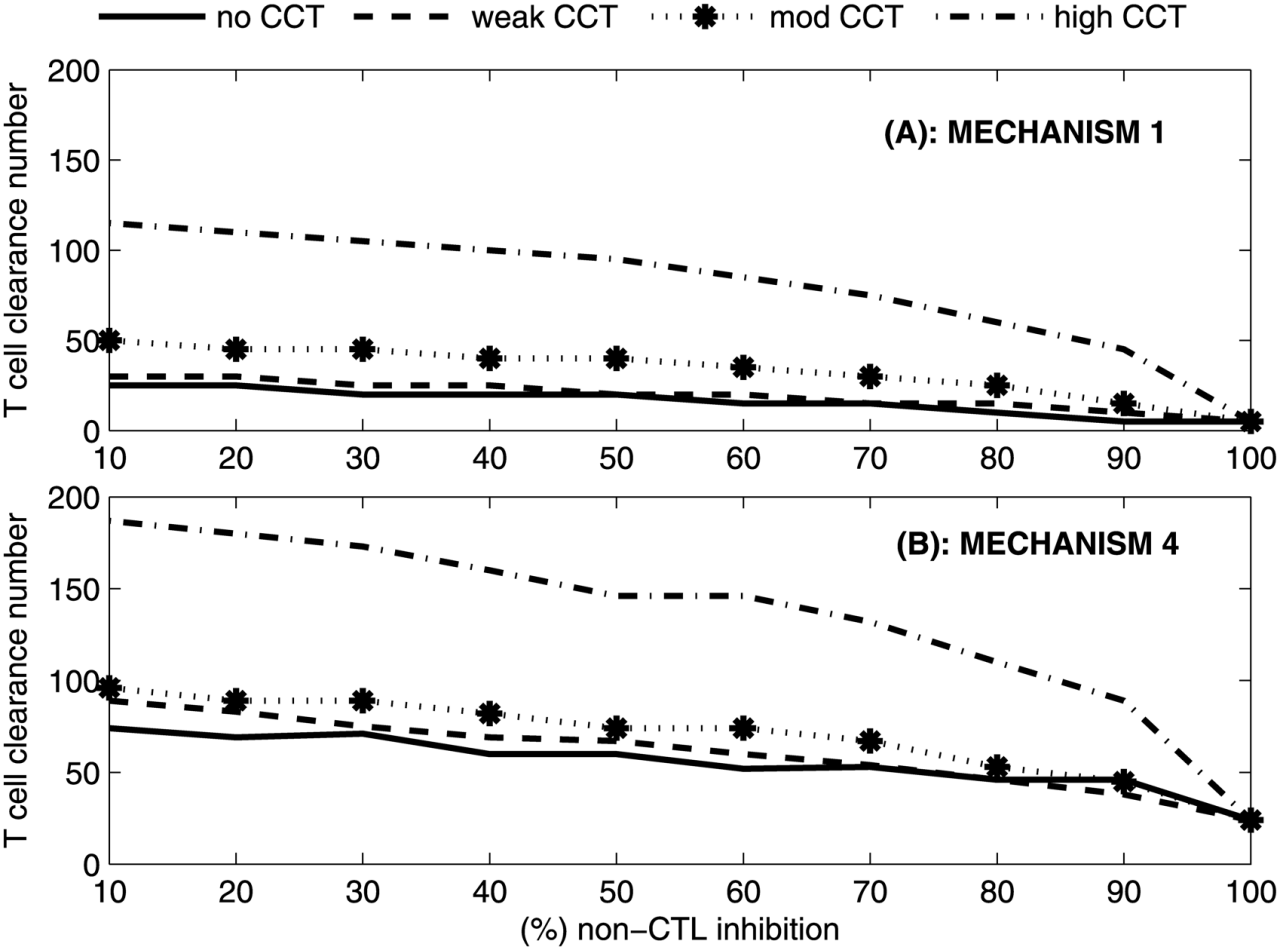
T cell clearance number under different CCT strengths. T cell clearance number (*n*_*CTL*_) reported for M1 and M4 under different strengths of CCT for a lattice size of *N* = 30^3^ hepatocytes.

Although the parameter *δ* is kept constant in M4, the number of cells cleared per day will change with the number of infected cells. The values of *n*_*CTL*_ in Fig 4D represent the number of cells killed at the peak of infection at day 45 and will be high, indeed substantially higher than for M1 which is assumed to remove a constant number of cells per day (Fig 5 and reported in S2–S8 Tables). However the number of cells killed per day under M4 will decrease as clearance occurs to the point where there is little remaining infection and therefore few cells killed per day at that later time. Hence the large discrepancy between *n*_*CTL*_ values. Nevertheless M4 achieves clearance with lower HT.

Despite stochasticity within the model, these results are robust and vary little from simulation to simulation, as they are conducted with a relatively large number of hepatocytes (S2 and S5 Tables).

### CCT can inhibit clearance of acute infection

Our simulations suggest CCT has an impact on clustering of infected cells in the establishment of acute infection. Now we calculate its role in impeding clearance of infection.

If CCT strength is moderate or high then the T cell clearance number increases substantially for all values of non-CTL inhibition (except for 100% efficacy in Fig 5). For example, at 10% non-CTL inhibition and under M1, the T cell clearance number is 25, 30, 50 and 115 for no CCT, weak CCT, moderate CCT and high CCT respectively (Fig 5). In another example, at 80% non-CTL inhibition and under M1, the T cell clearance number is 10, 15, 25 and 60 for no CCT, weak CCT, moderate CCT and high CCT respectively. We additionally observe that for lower efficacies of non-CTL inhibition, the T cell clearance number can achieve values greater than 100 particularly if CCT is on the stronger side. This implies that in the case of stronger CCT, one T cell would have to kill more infected cells than likely feasible in order to successfully achieve clearance of acute infection without excessive liver damage. The failure to achieve such high T cell clearance can result in chronic infection.

In the unlikely scenario of 100% non-CTL inhibition, an increase in CCT strength from weak to high causes no change in *n*_*CTL*_ (which remains at value 5) while HT increases from 0.8 to 0.9 (S2 and S8 Tables).

### Non-CTL inhibition is important for clearance of acute infection, particularly under high CCT

In general, lower levels of non-CTL inhibition are associated with higher T cell clearance numbers for all strengths of CCT (Fig 5). As might be expected, the T cell clearance number for different levels of non-CTL inhibition shows that the number of cells required to be killed by each CTL per day decreases as the impact of non-CTL processes increases (Figs 4 and 5). For example, at 10% non-CTL inhibition and under high CCT, the T cell clearance number is high at 115 and 187 under M1 and M4 respectively. This indicates that one T cell is required to kill 115 and 187 infected cells per day under M1 and M4 respectively which seems infeasible (see Discussion). The value of *n*_*CTL*_ decreases to more plausible values of 45 and 89 infected cells per day under M1 and M4 respectively if non-CTL inhibition efficacy increases to 90%. Therefore higher levels of cytokine production are required for infection clearance under feasible T cell clearance numbers, particularly when CCT strength is on the higher side. However higher levels of non-CTL inhibition are insufficient to clear infection on their own irrespective of CCT strength, in line with previous results [13]. We also observe a decrease in HT and viremia clearance time (*t*_*v*_) upon an increase in the efficacy of non-CTL inhibition for a constant T cell clearance number (S7 Table), which is consistent with previous results [13, 39].

### CCT can inhibit the impact of neutralizing antibodies but not of refractory cells

In the past, neutralizing antibodies and refractory cells have been hypothesized as expediting clearance of acute HBV infection [14, 15]. Refractory cells achieve this by not supporting reinfection and viral replication while passing these properties to daughter cells upon proliferation; neutralizing antibodies clear infectious virions [13–15]. To determine whether CCT can impact these mechanisms, we simulate our model assuming that these two mechanisms are involved in the immune mediated clearance along with CTL and non-CTL responses. Simulations are performed only for extreme non-CTL inhibition values (10% and 90%) under mechanism M1 and assuming that 2×10^8^ T cells are available every day. The effect of neutralizing antibodies (after day 40) is simulated using the relation t_1/2_ = AV^0.59^, where A = 3.9x10^-7^ is calculated assuming a 4 hr half-life of HBV virions at day 40 [40].

If refractory cells occur, then *n*_*CTL*_ remains unchanged (and stays at the lowest possible value of 5) irrespective of the level of CCT and non-CTL inhibition (S9 Table). So CCT will have little impact in the presence of refractory cells. In contrast, *n*_*CTL*_ varies with the level of CCT and non-CTL inhibition when we include neutralizing antibodies (S9 Table). For example, at 10% non-CTL inhibition, *n*_*CTL*_ takes value 15, 45 and 90 at weak, moderate and high CCT respectively. Therefore CCT can substantially impact the ability of neutralizing antibodies to clear infection.

### Incidence of fulminant hepatitis is associated with CCT strength, T cell response and cytokine levels

Regeneration of the liver is very rapid; the liver usually returns to a normal size in 1 to 2 weeks following the removal of greater than 50% of the liver mass [41]. Therefore, HT>6 within 12 weeks can be considered as a destructive process (which for these simulations we denote as fulminant hepatitis). As very little is known about what causes fulminant hepatitis, we explore what combinations of T cell response, non-CTL inhibition and CCT strength can result in such high levels of liver destruction. For this investigation, we vary T cell response and record HT for different combinations of non-CTL inhibition and CCT strength, employing M1 as a surrogate for the more biologically relevant M3, and assuming that 2×10^8^ T cells are available every day.

**Fig 6:**
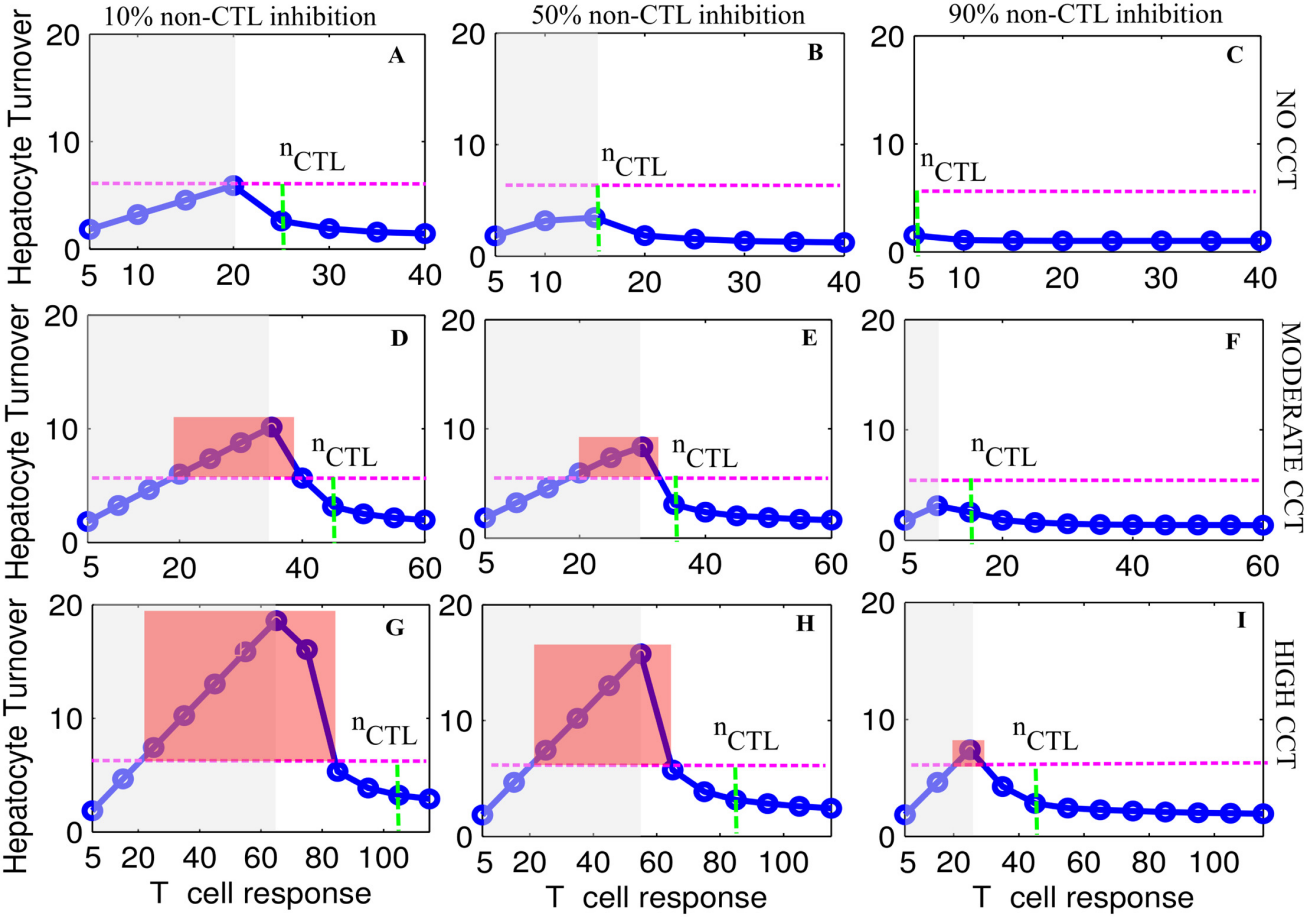
CCT, non-CTL inhibition and fulminant hepatitis. The level of hepatocyte turnover within 12 weeks after the peak of acute infection as a function of T cell response at different non-CTL inhibition levels and CCT strength. Subfigures (A, B and C) in row 1 represent the case of no CCT. Similarly rows 2 and 3 represent moderate and high CCT respectively while columns 1, 2 and 3 represent the case of 10%, 50% and 90% non-CTL inhibition respectively. Pink and green dashed line represent HT = 6 and *n*_*CTL*_ respectively while red blocks represent ranges of T cell response where fulminant hepatitis is most likely. Here, the grey area represents the range of T cell response which is inadequate to clear viremia within 12 weeks. All simulations were conducted using a lattice size of *N* = 30^3^ hepatocytes.

We find that fulminant hepatitis is less likely to arise if there is no CCT (absence of red blocks in Fig 6(A)–6(C)). Moderate CCT can result in fulminant hepatitis but only when cytokine levels are relatively low (Fig 6(D) and 6(E)). On the other hand high CCT can result in fulminant hepatitis even if cytokine levels are high (Fig 6(I)).

Another surprising observation is that only moderate T cell responses (away from the extreme values that result either in quick clearance (high T cell response) or little impact (low T cell response)) cause fulminant hepatitis (as shown by red blocks in Fig 6). The range of T cell response (the width of the red block) resulting in fulminant hepatitis varies with CCT strength and non-CTL inhibition efficacy. For example, this range is [20, 35] and [20, 40] at 10% and 50% non-CTL inhibition respectively for moderate CCT.

As both virus and host system are adaptive, they have the capability to upregulate or downregulate any machinery that supports or controls infection. If CCT increases over time while T cell response and non-CTL inhibition are kept constant, then this can also result in fulminant hepatitis. For example, while T cell response = 25 and non-CTL inhibition = 90%, an increase in CCT from none (Fig 6C) to high (Fig 6I) results in the possibility of fulminant hepatitis. Similarly if CCT remains constant but cytokine effectiveness decreases then fulminant hepatitis can result (Fig 6F compared to Fig 6D).

It is also noteworthy that fulminant hepatitis can occur both with clearance (white shaded regions) and with the failure of clearance (grey shaded regions).

In summary, it is CCT strength along with T cell response and efficacy of non-CTL inhibition which decides whether acute infection will result in clearance or non-clearance with or without the occurrence of fulminant hepatitis of the liver.

## Discussion

Chronicity usually represents the failure of the immune system to mount an effective response against acute HBV infection [33]. The pathogenesis of HBV is also dependent on several other factors such as the size of the inoculum, and host and viral factors [42, 43]. In order to improve the efficacy of existing treatment options for chronic hepatitis B infections, it is important to fully comprehend host and viral factors that are behind the establishment and clearance of acute infection.

The spread of HBV infection mainly occurs through infection from extracellular virions [3, 44]. Some experimental studies also indicate that CCT (or a similar mechanism) can support the spread of HBV infection [3, 44]. Our findings supported this hypothesis as experimentally observed clusters during early phases of infection were only observed in our simulations when CCT acts as a mechanism of the spread of HBV infection [3]. Our analysis also indicated that HBV is less likely to support CCT between two infected cells [8]. The liver is fully infected more quickly in the case of high CCT compared to its low counterpart. However, model simulations further showed that mean cccDNA copies/cell remain between 15 and 20 at the peak acute HBV infection level, irrespective of the inclusion or exclusion of CCT as a mechanism of viral spread. This could be a reason why CCT as a mechanism of HBV viral spread has not been generally considered in the past.

In contrast to its minimal impact on the establishment of acute infection, CCT hinders clearance and therefore can play an important role in T cell exhaustion and viral persistence. The T cell clearance number (*n*_*CTL*_) was lower under weak/zero CCT compared to moderate or high CCT. Since no or weak CCT can mimic a slower replicating virus, our results seem to be in line with the Whalley et al. observation that the slower replicating virus could induce a weaker CTL response [20].

As shown earlier, a high CCT results in faster viral spread and requires a higher T cell clearance number (≥96 for lower cytokine levels which seems infeasible and discussed later in detail) to achieve acute HBV infection clearance. Therefore, HBV under high CCT can possibly maintain high antigen loads despite the presence of a high number of T cells (in the feasible range of ≤96). Such persistent exposure of high antigen loads to T cells can lead to T cell exhaustion through functional T cell impairment and unresponsiveness [21, 45]. Whalley et al., hypothesized that enhanced viral replication can overcome the immune system [20]. Our simulations where enhanced viral replication is simulated by an increase in CCT strength supported this hypothesis [35]. Since this virus has already been found capable of inhibiting several host factors such as IFN-*β* production and blocking IFN-*α*-mediated pathways [42], it is possible that HBV may also have the capability to upregulate CCT strength to increase its chances of survival. Therefore if HBV exhibits CCT, then its chances of escaping the immune system increase, either by being less sensitive to the immune system [46] or putting too much burden on it. It will be worth investigating in the future if different HBV genotypes varied CCT strength which could also explain why they have different antiviral response rates [47].

Various dedicated reports and modelling studies have investigated the relative contribution of CTL and non-CTL mechanisms in immune-mediated clearance of HBV acute infection [13, 16, 23–25, 35, 42, 45]. Both CTL and non-CTL mechanisms, when acting alone, were insufficient to clear acute infection [13, 23]. Similar to Ciupe et al. [16], we also noted that acute HBV infection clearance relies on the combined strength of CTL and non-CTL components of the adaptive immune response (S1–S8 Tables). The feasibility of some of these combinations of CTL and non-CTL components can be constrained by physiological and other factors. For example, higher levels of non-CTL inhibition seem infeasible because IFN-*γ* suppresses hepatocyte proliferation which in turn can cause the liver to stop regenerating and functioning [48]. In addition to a high number of granules that can be replenished every 4 hours, T cells also have high diffusivity which allows a T cell to kill a large number of infected cells in a day (or, the T cell response to be quite high [49, 50]). However liver destruction of 30% per day or more is highly improbable [51] which limits the value of the T cell response to be less than 96 infected cells per day (96 infected cells per day killed by each of the 2×10^8^ T cells equates to ≈2×10^10^ of the 6×10^10^ hepatocytes). For the above reasons, an adaptive immune response with either T cell responses greater than 96 infected cells per day or non-CTL inhibition close to 100% can be considered as infeasible.

In clinical studies, patients who cleared acute HBV infection had a stronger immune response compared to those who did not clear infection [21]. More specifically, patients who resulted in non-clearance had lower levels of cytokines (indicated by lower IL-10 and TGF- *β* production) or T cell activity [45]. We also found that the T cell clearance number is significantly higher when non-CTL inhibition is low (Fig 5). In some cases (especially when CCT strength was high and non-CTL inhibition was on the lower side), the T cell clearance number crossed the threshold of infeasibility (*n*_*CTL*_≥96). Thus, in the absence of adequate cytokine production, high levels of T cell clearance number maintained over a period of time could also result in the exhaustion of HBV-specific T cells and non-clearance of infection [45]. Although regulatory cells can suppress HBV-specific T cells once chronic infection is established, it has been puzzling how the establishment of chronicity leads to a state of relative collapse of virus-specific T cells [45]. Our simulations suggested that the lower cytokine production could be a factor in T cell exhaustion and the establishment of chronicity. In general, a weaker immune response (either caused by a T cell response lower than the T cell clearance number or an inefficient cytokine production) and T cell exhaustion are among the major causes of non-clearance of acute infection. Our analysis showed that the adaptive immune response could always result in infection clearance independent of non-CTL inhibition with minimal T cell clearance number, if there are a high number of available T cells/day (simulated with 2×10^9^ T cells available every day in S7 Table). These results are in line with the observation that if the number of virus-specific CD8+ T cells is high then clearance is independent of cytokine levels [35].

Almost 1% of acute hepatitis infections develop fulminant hepatitis. Fulminant hepatitis is assumed to be caused by either faster replicating virus [20] or a higher T cell response [35]. Our simulations indicated that a high T cell response and enhanced viral replication are not the only two mechanisms that can contribute to the incidence of fulminant hepatitis. The level of non-CTL inhibition is also a major determinant along with T cell response and CCT strength. We additionally found that HBV infection that does not employ CCT as a mechanism of viral spread is less likely to experience fulminant hepatitis despite inadequate non-CTL inhibition and minimum T cell response.

Despite its usefulness, our model has some limitations. First of all, our model does not differentiate between CCT and the scenario where infection of an uninfected cell is due to virions exported from neighbouring infected cells. In addition, we did not explore the hypothesis that hepatocytes can lose the ability to replicate HBV, or to express HBV antigens, and so have a strong selective advantage [23].

In conclusion, our model provides insights into the process of viral establishment and dissects factors that can result in clearance, non-clearance or fulminant hepatitis of acute HBV infection. While CCT has no substantial impact on the establishment of infection, it can hinder its clearance. CCT can also be a factor in causing fulminant hepatitis (and non-clearance), particularly for a quantitatively suboptimal T cell number and non-CTL inhibition. Our results indicate that it is the combination of CCT strength, cytokine production and the T cell clearance number that decides the fate of HBV acute infection.

## Supporting Information

S1 Fig Infection on a 3-D lattice there is no cell-to-cell transmission. Infected cells shown in red dots on a 3D lattice at day 12 when there is no cell-to-cell transmission (CCT).
(TIF)

S2 Fig Infection on a 3-D lattice there is moderate cell-to-cell transmission. Infected cells shown in blue dots on a 3D lattice at day 12 when there is a moderate cell-to-cell transmission (CCT) between infected cells and uninfected cells.
(EPS)

S3 Fig Change in cccDNA copies/cell over time with CCT strength. Mean cccDNA copies/cell during the establishment of acute HBV infection for different CCT strengths under the assumption that there is no CCT between two already infected cells.
(EPS)

S4 Fig HT reported for different strengths of CCT and non-CTL inhibition. How different cytolytic killing mechanisms perform under different strengths of CCT and non-CTL inhibition for the T cell clearance number (*n*_*CTL*_) reported for M1, M2, M3 and M4 in Fig 5 in the main text. Each experiment was repeated 100 times, and then mean and 95% confidence interval of hepatocyte turnover (HT) was reported. All simulations were conducted using a lattice size of N = 30^3^ hepatocytes.
(EPS)

S1 Table The relative contribution of superinfection and amplification in the cccDNA accumulation at day 45. Contribution of superinfection (from free virions and cell-to-cell transmission (CCT)) and amplification in the cccDNA accumulation at day 45 in simulations with 27,000 hepatocytes.
(DOCX)

S2 Table Results under CTL mechanisms M1 to M4 assuming no CCT for a 3-D lattice of 27,000 cells. All mechanisms assume that 2×10^8^ CTL are available every day. In each case 100 simulations are conducted and then mean (min-max) are reported. For the performance of M1 to M3, we first determine T cell clearance number (*n*_*CTL*_) for M1 and then use the same value for M2 and M3. On the other hand, M4 is adaptive and is moderated by the number of infected cells. The results for mechanism 4 are derived after determining the minimum delta that can achieve clearance. Clearance is defined as achieving less than 1 copy of viremia in the body as well as zero cccDNA within 12 weeks starting after day 45 with HT≤3. NA represents non-availability of data.
(DOCX)

S3 Table Results under CTL mechanisms M1 to M4 assuming no CCT for a 3-D lattice of 10^6^ cells. All mechanisms assume that 2×10^8^ CTL are available every day. In each case 100 simulations are conducted and then mean (min-max) are reported. For the performance of M1 to M3, we first determine T cell clearance number (*n*_*CTL*_) for M1 and then use the same value for M2 and M3. On the other hand, M4 is adaptive and is moderated by the number of infected cells. The results for mechanism 4 are derived after determining the minimum delta that can achieve clearance. Clearance is defined as achieving less than 1 copy of viremia in the body as well as zero cccDNA within 12 weeks starting after day 45 with HT≤3. NA represents non-availability of data.
(DOCX)

S4 Table Results under CTL mechanisms M1 to M4 assuming weak CCT for a 3-D lattice of 27,000 cells. All mechanisms assume that 2×10^8^ CTL are available every day. In each case 100 simulations are conducted and then mean (min-max) are reported. For the performance of M1 to M3, we first determine T cell clearance number (*n*_*CTL*_) for M1 and then use the same value for M2 and M3. On the other hand, M4 is adaptive and is moderated by the number of infected cells. The results for mechanism 4 are derived after determining the minimum delta that can achieve clearance. Clearance is defined as achieving less than 1 copy of viremia in the body as well as zero cccDNA within 12 weeks starting after day 45 with HT≤3. NA represents non-availability of data.
(DOCX)

S5 Table Results under CTL mechanisms M1 to M4 assuming weak CCT for a 3-D lattice of 10^6^ cells. All mechanisms assume that 2×10^8^ CTL are available every day. In each case 100 simulations are conducted and then mean (min-max) are reported. For the performance of M1 to M3, we first determine T cell clearance number (*n*_*CTL*_) for M1 and then use the same value for M2 and M3. On the other hand, M4 is adaptive and is moderated by the number of infected cells. The results for mechanism 4 are derived after determining the minimum delta that can achieve clearance. Clearance is defined as achieving less than 1 copy of viremia in the body as well as zero cccDNA within 12 weeks starting after day 45 with HT≤3. NA represents non-availability of data.
(DOCX)

S6 Table Results under CTL mechanisms M1 to M4 assuming moderate CCT for a 3-D lattice of 27,000 cells. All mechanisms assume that 2×10^8^ CTL are available every day. In each case 100 simulations are conducted and then mean (min-max) are reported. For the performance of M1 to M3, we first determine T cell clearance number (*n*_*CTL*_) for M1 and then use the same value for M2 and M3. On the other hand, M4 is adaptive and is moderated by the number of infected cells. The results for mechanism 4 are derived after determining the minimum delta that can achieve clearance. Clearance is defined as achieving less than 1 copy of viremia in the body as well as zero cccDNA within 12 weeks starting after day 45 with HT≤3. NA represents non-availability of data.
(DOCX)

S7 Table Results under CTL mechanisms M1 to M4 assuming moderate CCT for a 3-D lattice of 27,000 cells. All mechanisms assume that 2×10^9^ CTL are available every day. In each case 100 simulations are conducted and then mean (min-max) are reported. For the performance of M1 to M3, we first determine T cell clearance number (*n*_*CTL*_) for M1 and then use the same value for M2 and M3. On the other hand, M4 is adaptive and is moderated by the number of infected cells. The results for mechanism 4 are derived after determining the minimum delta that can achieve clearance. Clearance is defined as achieving less than 1 copy of viremia in the body as well as zero cccDNA within 12 weeks starting after day 45 with HT≤3. NA represents non-availability of data.
(DOCX)

S8 Table Results under CTL mechanisms M1 to M4 assuming high CCT for a 3-D lattice of 27,000 cells. All mechanisms assume that 2×10^8^ CTL are available every day. In each case 100 simulations are conducted and then mean (min-max) are reported. For the performance of M1 to M3, we first determine T cell clearance number (*n*_*CTL*_) for M1 and then use the same value for M2 and M3. On the other hand, M4 is adaptive and is moderated by the number of infected cells. The results for mechanism 4 are derived after determining the minimum delta that can achieve clearance. Clearance is defined as achieving less than 1 copy of viremia in the body as well as zero cccDNA within 12 weeks starting after day 45 with HT≤3. NA represents non-availability of data.
(DOCX)

S9 Table Results associated with neutralizing antibodies and refractory cells in addition to CTL and non-CTL response for a 3-D lattice of 27,000 cells. For simulations, we assume CTL mechanism M1 and that 2×10^8^ CTL are available every day and then determine T cell clearance number (*n*_*CTL*_). In each case 100 simulations are conducted and then mean (min-max) are reported.
(DOCX)
