# Supplementary figures and images for: Modelling the Impact of Cell-To-Cell Transmission in Hepatitis B Virus
Source: PLoS One. 2016 Aug 25;11(8):e0161978. doi: 10.1371/journal.pone.0161978 (PMC4999077; doi:10.1371/journal.pone.0161978)

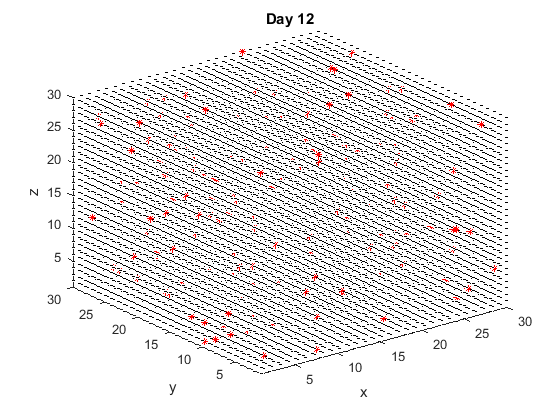

Supplement: S1 Fig — Infected cells shown in red dots on a 3D lattice at day 12 when there is no cell-to-cell transmission (CCT). (TIF) [file pone.0161978.s001.tif]
