# Supplementary material for: Modelling the Impact of Cell-To-Cell Transmission in Hepatitis B Virus
Source: PLoS One. 2016 Aug 25;11(8):e0161978. doi: 10.1371/journal.pone.0161978 (PMC4999077; doi:10.1371/journal.pone.0161978)
Supplement: S1 Table — Contribution of superinfection (from free virions and cell-to-cell transmission (CCT)) and amplification in the cccDNA accumulation at day 45 in simulations with 27,000 hepatocytes. (DOCX) [file pone.0161978.s005.docx]

|  | Superinfection (cccDNA) | Amplification (cccDNA) | (%)Amplification in the cccDNA accumulation |
| --- | --- | --- | --- |
| NO CCT | 18494 | 387753 | 95.5% |
| Weak CCT | 34720 | 463812 | 93.0% |
| MOD CCT | 28358 | 537766 | 95.0% |
| HIGH CCT | 32239 | 390226 | 92.3% |
