# Supplementary material for: Modelling the Impact of Cell-To-Cell Transmission in Hepatitis B Virus
Source: PLoS One. 2016 Aug 25;11(8):e0161978. doi: 10.1371/journal.pone.0161978 (PMC4999077; doi:10.1371/journal.pone.0161978)
Supplement: S6 Table — All mechanisms assume that 2×10^8^ CTL are available every day. In each case 100 simulations are conducted and then mean (min-max) are reported. For the performance of M1 to M3, we first determine T cell clearance number (nCTL) for M1 and then use the same value for M2 and M3. On the other hand, M4 is adaptive and is moderated by the number of infected cells. The results for mechanism 4 are derived after determining the minimum delta that can achieve clearance. Clearance is defined as achieving less than 1 copy of viremia in the body as well as zero cccDNA within 12 weeks starting after day 45 with HT≤3. NA represents non-availability of data. (DOCX) [file pone.0161978.s010.docx]

| Mechanism | Non-CTL inhibition | Cytolytic killing strength (delta) | **$n_{CTL}$** at the peak of infection | Hepatocyte Turnover (HT) [mean(min-max)] | Viremia Clearance time  ($t_{v}$ , days) [mean(min-max)] |
| --- | --- | --- | --- | --- | --- |
| 1  2  3  4 | 10% | NA  NA  NA  0.65 | 50  50  50  96 | 2.5 [2.5-2.6]  1.5 [1.5-1.5]  1.6 [1.5-1.6]  2.5 [2. 5-2.6] | 18 [17–19]  12 [11–13]  12 [11–13]  65 [52-84] |
| 1  2  3  4 | 20% | NA  NA  NA  0.60 | 45  45  45  89 | 2.8 [2.7-2.9]  1.6 [1.6-1.6]  1.6 [1.6-1.7]  2.7 [2.7-2.8] | 22 [21–23]  13 [12–14]  14 [13–15]  74 [59-84] |
| 1  2  3  4 | 30% | NA  NA  NA  0.60 | 45  45  45  89 | 2.5 [2.4-2.6]  1.5 [1.5-1.5]  1.6 [1.5-1.6]  2.4 [2.4-2.5] | 20 [19–21]  12 [12–13]  13 [12–14]  67 [54–84] |
| 1  2  3  4 | 40% | NA  NA  NA  0.55 | 40  40  40  82 | 2.8 [2.7-2.8]  1.6 [1.6-1.6]  1.6 [1.6-1.7]  2.5 [2.5-2.6] | 23 [22–24]  14 [14–16]  15 [14–16]  72 [60-84] |
| 1  2  3  4 | 50% | NA  NA  NA  0.50 | 40  40  40  74 | 2.4 [2.3-2.4]  1.5 [1.4-1.5]  1.5 [1.5-1.5]  2.6 [2.5-2.6] | 20 [20–22]  13 [13–15]  14 [13–15]  77 [61-84] |
| 1  2  3  4 | 60% | NA  NA  NA  0.50 | 35  35  35  74 | 2.5 [2.4-2.5]  1.5 [1.5-1.5]  1.6 [1.5-1.6]  2.2 [2.1-2.2] | 23 [23–25]  15 [15–17]  16 [15–17]  64 [48-84] |
| 1  2  3  4 | 70% | NA  NA  NA  0.45 | 30  30  30  67 | 2.5 [2.4-2.6]  1.5 [1.5-1.6]  1.6 [1.6-1.6]  2.1 [2.0-2.1] | 27 [26–28]  18 [17–19]  18 [17–19]  68 [52-84] |
| 1  2  3  4 | 80% | NA  NA  NA  0.35 | 25  25  25  53 | 2.3 [2.3-2.3]  1.5 [1.5-1.5]  1.6 [1.5-1.6]  2.2 [2.1-2.2] | 30 [28–32]  21 [19–22]  21 [20–22]  80 [70-84] |
| 1  2  3  4 | 90% | NA  NA  NA  0.30 | 15  15  15  45 | 2.5 [2.4-2.6]  1.6 [1.6-1.7]  1.7 [1.7-1.7]  1.7 [1.7-1.8] | 50 [48–52]  33 [32–34]  35 [34–37]  71 [58-84] |
| 1  2  3  4 | 100% | NA  NA  NA  0.15 | 5  5  5  24 | 0.8 [0.8-0.8]  0.8 [0.8-0.8]  0.8 [0.8-0.8]  1.1 [1.1-1.1] | 25 [22–29]  25 [22–28]  25 [22–28]  22 [20-26] |
