# Supplementary material for: Modelling the Impact of Cell-To-Cell Transmission in Hepatitis B Virus
Source: PLoS One. 2016 Aug 25;11(8):e0161978. doi: 10.1371/journal.pone.0161978 (PMC4999077; doi:10.1371/journal.pone.0161978)
Supplement: S9 Table — For simulations, we assume CTL mechanism M1 and that 2×10^8^ CTL are available every day and then determine T cell clearance number (nCTL). In each case 100 simulations are conducted and then mean (min-max) are reported. (DOCX) [file pone.0161978.s013.docx]

| **CTL and non-CTL response with neutralizing antibodies** | | | | |
| --- | --- | --- | --- | --- |
| CCT | Non-CTL inhibition | **$n_{CTL}$** | Hepatocyte Turnover (HT)  [mean(min-max)] | Viremia Clearance time ($t_{v}$ , days)  [mean(min-max)] |
| Weak | 10% | 15 | 2.5 [2.5-2.6] | 40 [28–51] |
| Weak | 90% | 5 | 1.0 [1.0-1.0] | 40 [28–56] |
| Moderate | 10% | 45 | 2.4 [2.4-2.5] | 12 [7–18] |
| Moderate | 90% | 15 | 2.1 [2.1-2.2] | 25 [8–43] |
| High | 10% | 90 | 3.0 [2.9-3.0] | 4 [2–9] |
| High | 90% | 35 | 2.5 [2.5-2.6] | 5 [2–8] |
| **CTL and non-CTL response with emergence of refractory cells** | | | | |
| CCT | Non-CTL inhibition | **$n_{CTL}$** | Hepatocyte Turnover (HT)  [mean(min-max)] | Viremia Clearance time ($t_{v}$ , days)  [mean(min-max)] |
| Weak | 10% | 5 | 1.2 [1.2-1.3] | 68 [65–71] |
| Weak | 90% | 5 | 0.8 [0.8-0.8] | 45 [43–46] |
| Moderate | 10% | 5 | 1.1 [1.1-1.2] | 62 [60–71] |
| Moderate | 90% | 5 | 0.9 [0.8-0.9] | 48 [46–50] |
| High | 10% | 5 | 1.8 [1.7-1.8] | 41 [7–84] |
| High | 90% | 5 | 0.9 [0.8-1.0] | 9 [2–28] |
| **CTL and non-CTL response with both neutralizing antibodies and emergence of refractory cells** | | | | |
| CCT | Non-CTL inhibition | **$n_{CTL}$** | Hepatocyte Turnover (HT)  [mean(min-max)] | Viremia Clearance time ($t_{v}$ , days)  [mean(min-max)] |
| Weak | 10% | 5 | 1.1 [1.0-1.1] | 45 [31–61] |
| Weak | 90% | 5 | 0.8 [0.7-0.8] | 32 [22–43] |
| Moderate | 10% | 5 | 1.1 [1.0-1.1] | 47 [32–59] |
| Moderate | 90% | 5 | 0.8 [0.8-0.9] | 32 [21–47] |
| High | 10% | 5 | 1.8 [1.7-1.8] | 42 [6–80] |
| High | 90% | 5 | 0.8 [0.8-0.9] | 8 [2–25] |
